# Isolation and Identification of the Five Novel Flavonoids from *Genipa americana* Leaves

**DOI:** 10.3390/molecules23102521

**Published:** 2018-10-02

**Authors:** Larissa Marina Pereira Silva, Jovelina Samara Ferreira Alves, Emerson Michell da Silva Siqueira, Manoel André de Souza Neto, Lucas Silva Abreu, Josean Fechine Tavares, Dayanne Lopes Porto, Leandro de Santis Ferreira, Daniel Pecoraro Demarque, Norberto Peporine Lopes, Cícero Flávio Soares Aragão, Silvana Maria Zucolotto

**Affiliations:** 1 Research Group on Bioactive Natural Products (PNBio), Laboratory of Pharmacognosy, Department of Pharmacy, Federal University of Rio Grande do Norte (UFRN), Natal 59010-180, Brazil; larissamarinaps@gmail.com (L.M.P.S.); jsfa.farma@gmail.com (J.S.F.A.); siqueira_emerson@hotmail.com (E.M.d.S.S.); rufinim@gmail.com (M.A.d.S.N.); 2 Multiuser Laboratory of Characterization and Analysis (LMCA), Federal University of Paraíba, João Pessoa 58051-900, Brazil; lucas.abreu@ltf.ufpb.br (L.S.A.); josean@ltf.ufpb.br (J.F.T.); 3 Laboratory of Quality Control of Medications (LCQMed), Department of Pharmacy, Federal University of Rio Grande do Norte (UFRN), Natal 59010-180, Brazil; daylopesporto@hotmail.com (D.L.P.); lean_sf@yahoo.com.br(L.d.S.F.); cicero.aragao@yahoo.com.br (C.F.S.A.); 4 Nucleus Research in Natural and Synthetic Products (NPPNS), Department of Physics and Chemistry, Faculty of Pharmaceutical Sciences of Ribeirão Preto, University of São Paulo, São Paulo 14040-903, Brazil; dpdemarque@gmail.com (D.P.D.); npelopes@fcfrp.usp.br (N.P.L.)

**Keywords:** Rubiaceae, jenipapo, HPLC-ESI-IT-MS/MS, flavonoids glycosides

## Abstract

*Genipa americana* is a medicinal plant popularly known as “jenipapo”, which occurs in Brazil and belongs to the Rubiaceae family. It is a species widely distributed in the tropical Central and South America, especially in the Cerrado biome. Their leaves and fruits are used as food and popularly in folk medicine to treat anemias, as an antidiarrheal, and anti-syphilitic. Iridoids are the main secondary metabolites described from *G. americana*, but few studies have been conducted with their leaves. In this study, the aim was to chemical approach for identify the main compounds present at the extract of *G. americana* leaves. The powdered leaves were extracted by maceration with EtOH: water (70:30, *v*/*v*), following liquid-liquid partition with petroleum ether, chloroform, ethyl acetate and *n*-butanol. A total of 13 compounds were identified. In addition three flavonoids were isolated from the ethyl acetate fraction: *quercetin-3-O-robinoside* (**GAF 1**), *kaempferol-3-O-robinoside* (**GAF 2**) and *isorhamnetin-3-O-robinoside* (**GAF 3**) and, from *n*-butanol fraction more two flavonoids were isolated, *kaempferol-3-O-robinoside-7-O-rhamnoside* (robinin) (**GAF 4**) and *isorhamnetin-3-O-robinoside-7-rhamnoside* (**GAF 5**). Chemical structures of these five flavonoids were elucidated using spectroscopic methods (MS, ^1^H and ^13^C-NMR 1D and 2D). These flavonoids glycosides were described for the first time in *G. americana*.

## 1. Introduction

*Genipa americana* L. belongs to Rubiaceae family, which contains approximately 140 genera and 2120 species occurring and in various places around the world, mainly in tropical and subtropical regions [1,2]. *Genipa americana* L. is a native Brazilian plant, with perennial foliage and widely distributed in tropical Central and South America, including the Cerrado biome [3,4,5]. It is popularly known as “jenipapo”, which comes from the indigenous language Tupi-Guarani and means “fruit used for painting” [6]. Its fruits are usually processed for liqueurs, candies, and ice cream preparations [7], and are used as tonic against anemia [8]. Ethnopharmacological studies report that different parts of the species have been used in folk medicine, including leaves as an antidiarrheal and anti-syphilitic (decoction) [9]; leaf preparation for treatment of fevers (macerated) [10]; and to treat liver diseases [8].

Most of the phytochemical and pharmacological studies reported to *G. americana* were conducted with its fruits [4]. Regarding pharmacological studies especially with its leaves there are reports about anti-inflammatory [11], antiangiogenic [12], antidiarrheal, and anti-syphilis activity [9], inhibition of larval development and eclosion of sheep gastrointestinal nematodes [13], antidiabetic [14], and anticonvulsant effects [15].

Concerning the phytochemical composition, it has been reported to *G. americana* leaves the presence of secondary metabolites such as tannins [13], monoterpenes [16], and flavonoids [17], being iridoids the main secondary metabolites described until this moment [4,16,18,19,20,21]. Mainly for the leaves, the presence of following iridoids was already described: geniposidic acid [22], genipatriol [23], genamesides A–D [19], and our group recently isolated and identified the iridoids 1-hydroxy-7-(hydroxymethyl)-1,4a*H*,5*H*,7a*H*-cyclopenta[*c*]pyran-4-carbaldehyde, and 7-(hydroxymethyl)-1-methoxy-1*H*,4a*H*,5*H*,7a*H*-cyclopenta[*c*]pyran-4-carbaldehyde [24].

Meanwhile, although flavonoids are a large class of plant secondary metabolites widely distributed throughout the plant kingdom [24], that is structurally resembling in that most have a C_15_ phenyl-benzopyrone skeleton [25], the reports about its presence in *G. americana* are scarce, making it a topic that needs further investigation. Our research group detected the presence of flavonoids for the first time in *G. americana* leaves through a fingerprint by thin layer chromatography (TLC) using a specific reagent to detect flavonoids (NP reagent) [24].

In this context, the present study aimed to study the hydroethanolic extract (HE) obtained from leaves of *Genipa americana*, in terms of its phytochemical composition, especially with respect to flavonoid profiles obtained by UHPLC-DAD and LC-IT-ESI-MS/MS.

## 2. Results and Discussion

### 2.1. Extraction and TLC Analysis

Despite being a class of secondary metabolites that have shown promising biological activities in various studies [26], only our study described the presence of flavonoids in *G. americana* [24]. Therefore, the phytochemical investigation of this work was conducted to isolate and purify the flavonoids of *G. americana* leaf extract. Initially, the extract (HE) was fractioned and TLC analysis of the HE and fractions, with specific spray reagents, indicated the presence of flavonoids and iridoids [7,27]. The phytochemical screening of the HE, revealed with vanillin sulfuric acid, showed characteristics yellow spots (*R_f_* = 0.33 until 0.90), suggesting the presence of flavonoids as observed in our previous work [24]. Afterwards, a liquid-liquid extraction was carried out, affording the following fractions: petroleum ether (PE), chloroform (CHCl_3_), ethyl acetate (EtOAc), *n*-butanol (*n*-BuOH) and the residual aqueous fraction (RA). The PE fraction showed no spot in the TLC plate after spraying with vanillin sulfuric acid; CHCl_3_ fraction showed two purple spots (*R_f_* = 0.93 and 0.83), suggesting the presence of iridoids; the EtOAc fraction showed only one zone more evident (*R_f_* = 0.81 —purple), that indicated the presence of iridoids. When EtOAc fraction was revealed with Natural Product (NP) Reagent (UV 365 nm) a yellow zone was observed (*R_f_* = 0.55), suggesting the presence of flavonoids. TLC analysis of the *n*-BuOH fraction showed four yellow and orange spots (*R_f_* = 0.21 until 0.50—NP Reagent/UV 365 nm), suggestive of flavonoids [8].

### 2.2. UHPLC-DAD Characterization

The Research Group on Bioactive Natural Products (PNBio) has been studying the leaves of *G. americana* and in a recent publication [24] the presence of two new iridoids for its leaves has been identified, showing the chemical potential of the species.

As described earlier, most of the studies have described the presence of iridoids in the genus *Genipa*, being considered a chemotaxonomic marker [2,24]. Only our study identified flavonoids in leaves [24]. Furthermore, only one article described the presence of leucoanthocyanidins, catechins, and flavanones in the fruits of *G. americana* by HPLC analysis [28], and another study described its presence in fruits by colorimetric methods [13], demonstrating that the presence of flavonoids in leaf extract was not described previously.

**Figure 1:**
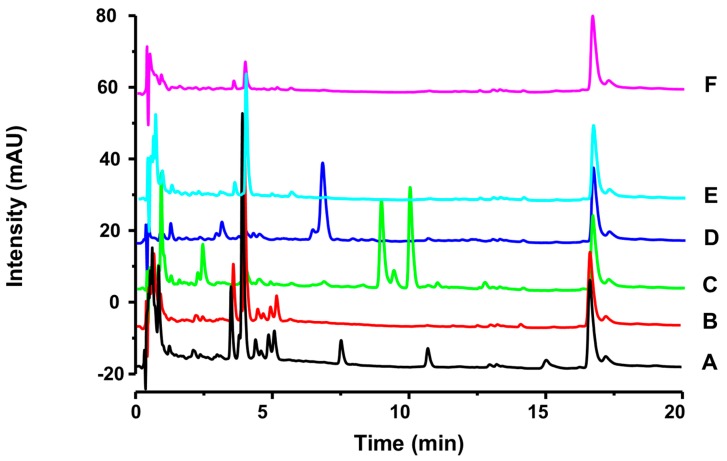
UHPLC-DAD chromatogram of *G. americana* leaf extract and fractions. Solvent system: mix of acetonitrile: water (with 0.3% of acetic acid); Flow rate: 0.3 mL/min; *Software* LC *Solution*; Wavelength: 254 nm. column C_18_ Shim-pack XR-ODS (Shimadzu^®^, Japan) (30 × 2 mm, 2.2 µm), the temperature 25 ± 2 °C. **A**: EtOAc fraction; **B**: *n*-BuOH fraction; **C**: CHCl_3_ fraction; **D**: PE fraction; **E**: Aqueous residual fraction; **F**: Extract HE.

Extract and fractions were analyzed by ultra-performance liquid chromatography (UHPLC). The chromatogram of the HE, recorded in 254 nm, showed four main peaks (3.5 min, UV: 246, 265 and 350 nm; 3.9 min, UV: 255 and 354 nm; 16.6 min, UV: 242 and 287 nm; 17.2 min, UV: 243 nm). Accordingly, the UV spectra of each peak observed in the chromatogram can suggest the presence the flavonoids and iridoids in HE of *G. americana* leaves (Figure 1).

### 2.3. HPLC-ESI-IT-MS/MS Characterization

Through the TLC and UHPLC-DAD, we can suggest the presence of iridoids and flavonoids in leaves extract from *G. americana*. Therefore, it was decided to perform an HPLC-MS/MS study to allow the identification these compounds.

In this way, we selected of HE, Fr.EtOAc, and Fr.*n*-BuOH to analyze. The HPLC-MS/MS revealed the presence of several majority peaks and allowed suggest the structures of 13 compounds—including five flavonoids (subsequently isolated), using the program DataAnalysis 4.2 (Bruker, Billerica, MA, USA) (Table 1).

*Coniferin* (MM = 342, C_16_H_22_O_8_, Table 1), showed signals at 342 [M − H]^−^; 179 [M − H − Glc]^−^. Already identified in *Ginkgo biloba* L. (Ginkgoaceae) [29].

*Asystasioside D* (MM = 536, C_22_H_32_O_15_, Table 1): showed signals at 559 [M + Na]^+^, 535 [M − H]^−^; 571 [M + Cl]^−^, 535, 373 [M − H − Glc]; 210 [M − H − Glc − Glc] [aglycone genipinic acid]. This compound is a iridoid glucoside and had already been described in *Asystasia bella* (Harv.) [30].

*Geniposidic acid* (MM = 374, C_16_H_22_O_10_, Table 1), showed signals at 397 [M + Na]^+^, 373 [M − H]^−^, 409 [M + Cl]^−^, 379 [M + Na − H_2_O]^+^, 217 [M + Na − H_2_O − Glc]^+^, 210 [M − H − Glc]^−^, 172 [M + Na − H_2_O − Glc − CO_2_]^+^, 166 [M − H − Glc − CO_2_]^−^, 148 [M − H − Glc − CO_2_ − H_2_O]^−^, 122 [M − H − Glc − C_3_H_4_O_3_]^−^. It is a iridoid already identified in fruit of *Gardenia jasminoides* Ellis (Rubiaceae) [19,22,31,32] and *Wendlandia formosana* Cowan leaves [33].

*Tarenoside* (MM = 358, C_16_H_25_O_9_, Table 1): showed signals at 357 [M − H]^−^; 393 [M + Cl]^−^, 194 [M − H − Glc]^−^. This compound is an iridoid already identified in *Wendlandia formosana* Cowan leaves and *Genipa americana* [2,33].

*Loganic acid* (MM = 376, C_16_H_24_O_10_, Table 1): showed signals at 375 [M − H]^−^; 213 [M − H − Glc]; 169, 125. It had already been described in *Anthocephalus chinensis* (Rubiaceae) and *Ophiorrhiza liukiuensis* (Rubiaceae) [2]. Loganic acid is described for the first time to *Genipa* genus.

*Chlorogenic acid* (MM = 354, C_16_H_18_O_9_, Table 1), showed signals at 355 [M + H]^+^, 353 [M − H]^−^, 162 [caffeic acid], 190 [quinic acid]. It had already been isolated in fruit of *Gardenia jasminoides* Ellis (Rubiaceae) [31,34], but the first time is described in the *Genipa* genus.

*Kaempferol-3-O-hexoside-deoxyhexoside-7-O-deoxyhexoside* (**GAF 4**, MM = 740, C_33_H_39_O_20_, Table 1), showed signals at 741 [M + H]^+^, 739 [M − H]^-^, 595, 593 [M − Glc]^−^, by scission of the robinose glycan residue to yield the radical anion 433 [M − Glc − Glc]^−^, 287; radical anion fragments by loss of the rhamnose glycan residue to yield the radical anion 285 [M − Glc − Glc − Glc]^−^ [25].

*Isorhamnetin-3-O-hexoside-deoxyhexoside-7-O-deoxyhexoside* (**GAF 5**, MM = 770, C_34_H_42_O_21_, Table 1), showed signals at 769 [M − H]^−^; 771 [M − H]^+^; 625 [M − Rha + H]^+^; 463; 623 [M − Rha − H]^−^; 317; 315 [M − Rha − Rha − Glc-H]^−^ [35].

*Quercetin-3-O-hexoside-deoxyhexoside* (**GAF 1**, MM = 610, C_27_H_30_O_16_, Table 1), showed signals at *m*/*z* 611 [M + H]^+^, 609 [M − H]^−^, 465, corresponding to the loss of rhamnose unit, 301 [M − Glc − Glc]^−^, corresponding to the loss of the rhamnosylgalactose unit, and 303. It is suggested that the sugar is of galactose isomerism, since this form was isolated, and plants usually produces a conformation of sugar glucose or galactose, not being the rutin.

*1,3-Di-O-caffeoylquinic acid* (cynarine, MM = 516, C_25_H_24_O_12_, Table 1): showed signals at 515 [M − H]^−^, 353 [chlorogenic acid]; 190 [acid quinic]; 178. This compound is a chlorogenic acid derivatives. It had already been isolated in *Cynara cardunculus* leaves [36].

*Teneoside A* (MM = 518, C_22_H_30_O_14,_ Table 1): showed signals at 519 [M + H]^+^, 373 [M − Ram + H]^+^, 211 [M − Ram − Glc + H]^+^, 517 [M − H]^−^, 371 [M − Ram − H]^–^. This is an iridoid glucoside, and it has already been isolated in *Hedyotis tenelliflora* Blume (Rubiaceae) [2], but is also described for the first time in the *Genipa* genus.

*Kaempferol-3-O-hexoside-deoxyhexoside* (**GAF 2**, MM = 594, C_27_H_30_O_16_, Table 1) showed signals at 595 [M + H]^+^, 593 [M − H]^−^, and 449 [M − Glc]^−^ due to the loss of rhamnose, and 288 [M − Glc]^−^ due to the loss of the rhamnosylgalactose unit. Likewise, it is suggested that the sugar is galactose, not being, therefore the *Kaempferol-3-O-rutinosideo*.

*Isorhamnetin-3-O-hexoside-deoxyhexoside* (**GAF 3**, MM = 624, C_28_H_33_O_16_, Table 1) showed signals at 625 [M + H]^+^, 623 [M − H]^−^, 479, 315, and 317 [37].

The present study contributed to describe a new phytochemical approach for *Genipa americana*, considering that until this moment most of the studies reported only the presence of iridoids [2,4,24].

### 2.4. Isolation of Major Compounds

In order to verify the positions of the sugars of the *O*-glycosides present in the flavonoids identified by HPLC-MS/MS (Section 2.3), the major constituents of the EtOAc and *n*-BuOH fractions were isolated and identified.

Based on the chromatographic profile observed by TLC and UHPLC analysis, EtOAc fraction (3.27 g) was chosen initially to be fractionated by vacuum liquid chromatography (VLC) and then fractions 8 and 9 were gathered and chromatographed by classical column using silica gel (0.063–0.200 mm) and Sephadex LH-20 gel, which yielded subfractions 134. Then subfraction (61–111) was gathered and isolated by preparative HPLC and three well-separated peaks were obtained in the chromatogram. Thereby, the following flavonoids were purified: *quercetin-3-O-robinoside* (**GAF 1**, 4.2 mg), *kaempferol-3-O-robinoside* (**GAF 2**, 4.0 mg) and *isorhamnetin-3-O-robinoside* (**GAF 3**, 6.0 mg).

Since the *n*-BuOH fraction (5.0 g) also had a flavonoid rich phytochemical profile (Figure 1) and higher yield, it was submitted to fractionation by VLC with silica gel, which yielded 11 subfractions. The fraction 4 (1.07 g) was isolated by classical column chromatography, and its sub-fraction 4-E of chromatography (1.0 g) was dissolved in methanol and chilled to 2 °C, for five days, which promoted the crystallization of a part of the sample, allowed its separation. These fractions were named fraction 4-E.1 (crystallized) and fraction 4-E.2 (not crystallized). The fraction 4-E.1 was submitted to preparative HPLC and two flavonoids were further separated: *kaempferol-3-O-robinoside-7-O-rhamnoside* (robinin) (**GAF 4**, 10.0 mg) and *isorhamnetin-3-O-robinoside-7-O-rhamnoside* (**GAF 5**, 9.0 mg). Under the above conditions, a satisfactory separation of targeted compounds was obtained.

**Figure 2:**
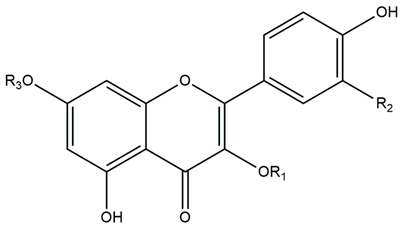
Chemical structures of flavonoids **GAF 1**–**5** isolated from the leaves of *Genipa americana*.

The chemical structures of these five compounds were elucidated by MS and 1D and 2D ^1^H- and ^13^C-NMR spectroscopic analyses. Comparison with literature data allowed to confirm the structures of the compounds **GAF 1**, **2**, **3**, **4**, and **5** as flavonoids (Figure 2).

In the study, a simple and effective procedure allowed the isolation of flavonoids from the leaves of *G. americana*. Previously only two papers described the detection of flavonoids in fruits [13,28] and one study published by our group identified these compounds in leaves [24] from *G. americana*. It is important to mention that this is a new phytochemical approach concerning the leaves of this species. Flavonoids are products of secondary metabolism in plants and are of interest to the pharmaceutical and food industries because of their reported wide range of biological effects [26], and they have long been associated with good health benefits, which could be attributed to their antioxidant capabilities [26].

### 2.5. Identification of the Isolated Compounds

To confirm the positions of the sugars of the *O*-glycosylated flavonoids identified by HPLC-MS/MS, major compounds from EtOAc and *n*-BUOH fractions were isolated, obtaining five flavonoids. Their structures were identified by comparison of their spectroscopic data reported, including ESI-MS and NMR data.

*Quercetin 3-O-robinoside* [38] (**GAF 1**, Figure 2): λ_max_ 255, 366 nm. It was assigned with a molecular formula C_27_H_30_O_16_ of ESI-MS, *m*/*z* 609.1403 [M − H]^−^. ^1^H-NMR (400 MHz, DMSO-*d*_6_) *δ*: 7.66 (1H, dd, *J* = 8.5, 2. 2 Hz, H-6′), 7.53 (1H, d, *J* = 2.2 Hz, H-2′), 6.82 (1H, d, *J* = 8.5 Hz, H-5′), 6.39 (1H, s, H-8), 6.19 (1H, s, H-6), 5.32 (1H, d, *J* = 7.7 Hz, galactosyl H-1′′), 4.42 (1H, d, *J* = 1.3 Hz, rhamnosyl H-1′′′), 3.10 (2H, t, *J* = 9.5 Hz, H-4′′′), 1.07 (3H, d, *J* = 6.2 Hz, H-6′′′, rhamnosyl-Me). ^13^C-NMR (100 MHz, DMSO-*d*_6_) *δ*: 177.35 (C-4), 164.25 (C-7), 161,20 (C-5), 156.38 (C-9), 156.26 (C-2), 148.56 (C-4′), 144.85 (C-3′), 133.47 (C-3), 121.93 (C-6′), 121.03 (C-1′), 115.96 (C-2′), 115.19 (C-5′), 103.77 (C-10), 102.07 (C-1′′), 99.99 (C-1′′′), 98.80 (C-6), 93.58 (C-8), 73.54 (C-5′′), 73.06 (C-3′′), 71.92 (C-4′′′), 71.10 (C-2′′), 70.62 (C-3′′′), 70.43 (C-2′′′), 68.28 (C-5′′′), 68.04 (C-4′′), 65.09 (C-6′′), 17.92 (C-6′′′). The ^13^C-NMR spectrum showed the sugars to be in the β-d-galactopyranose and α-l-rhamnopyranose forms.

The compounds **GAF 1** has already been identified in: *Alternanthera brasiliana* (L.) Kuntze (Amaranthaceae) [37]; *Strychnos variabilis* (Loganiaceae) leaves; *Robinia pseudacacia* (Fabaceae) fruits; *Lespedeza hedysaroides* (Fabaceae) epigeal part; *Costus sanguineus* (Costaceae)leaves; *Crataegus pinnatifida* (Rosaceae) flowers; *Brickellia chlorolepis* (Asteraceae) leaves [38]; *Lysimuchia vulgaris* (Primulaceae) [39]; and *Aspalathus linearis* (Fabaceae) [40]. No reports were found for the Rubiaceae family.

*Kaempferol-3-O-robinoside* [38,41] (**GAF 2**, Figure 2): λ_max_ 265, 355 nm. It was assigned with a molecular formula C_27_H_30_O_16_ by ESI-MS, *m*/*z* 593.1508 [M − H]^−^. ^1^H-NMR (400 MHz, DMSO-*d*_6_) *δ*: 8.05 (2H, d, *J* = 8.8 Hz, H-2′, H-6′), 6.86 (2H, d, *J* = 8.8 Hz, H-3′, H-5′), 6.41 (1H, s, H-8), 6.19 (1H, s, H-6), 5.31 (1H, d, *J* = 7.7 Hz, galactosyl H-1′′), 4.40 (1H, s, rhamnosyl H-1′′′), 3.09 (1H, t, *J* = 9.5 Hz, H-4′′′), 1.06 (2H, d, *J* = 6.1 Hz, H-6′′′). ^13^C-NMR (100 MHz, DMSO-*d*_6_) *δ*: 177.39 (C-4), 164.58 (C-7), 161.18 (C-5), 159.97 (C-3′), 156.54 (C-2), 156.47 (C-9), 149.41 (C-4′), 133.29 (C-3), 130.96 (C-6′), 121.2 (C-2′), 120.85 (C-1′), 115.07 (C-5′), 103.79 (C-10), 100.06 (C-1′′′), 102.07 (C-1′′), 98.85 (C-6), 93.79 (C-8), 73.52 (C-5′′), 72.97 (C-3′′), 71.90 (C-4′′′), 71.10 (C-2′′), 70.61 (C-2′′′), 70.42 (C-3′′′), 68.29 (C-5′′′), 68.00 (C-4′′), 65.30 (C-6′′), 17.93 (C-6′′′).

The compounds **GAF 2** has already been identified in: *Strychnos variabilis* (Loganiaceae) leaves; *Atropa beladona* (Solanaceae) leaves [38]; *Blackstonia perfoliata* (L.) (Gentianaceae) aerial parts [42]; *Gynura formosana* Kitam. (Asteraceae) [43]; *Astragalus tana* Sosn. (Fabaceae) [37]; *Caragana chamlagu* Lam. (Fabaceae) [37]; *Alternanthera brasiliana* (L.) Kuntze (Amaranthaceae) [37]; and *Rumex chalepensis* Mill (Polygonaceae) [41]. No reports were found for the *Genipa* genus.

*Isorhamnetin-3-O-robinoside* [37] (**GAF 3**, Figure 2): λ_max_ 255, 370 nm. It was assigned with a molecular formula C_28_H_33_O_16_ by ESI-MS, *m*/*z* 623.1596 [M − H]^−^. ^1^H-NMR (400 MHz, DMSO-*d*_6_) *δ*: 8.00 (1H, d, *J* = 2.3 Hz, H-2′), 7.51 (1H, dd, *J* = 8.4, 2.1 Hz; H-6′), 6.90 (1H, d, *J* = 8.3 Hz, H-5′), 6.42 (1H, s, H-8), 6.19 (1H, d, *J* = 1.8 Hz, H-6), 5.45 (1H, d, *J =* 7.7 Hz; H-1′′), 4.42 (1H, d, *J* = 1.7 Hz; H-1′′′), 3.85 (3H, s, H-3′-O-CH_3_), 3.08 (2H, t, *J* = 9.4 Hz, H-4′′′), 1.05 (3H, d, *J =* 6.1 Hz, H-6′′′). ^13^C-NMR (100 MHz, DMSO-*d*_6_) *δ*: 177.29 (C-4), 164.65 (C-7), 161.19 (C-5), 156.46 (C-9), 156.33 (C-2), 149.45 (C-4′), 146.99 (C-3′), 133.08 (C-3), 121.96 (C-6′), 121.05 (C-1′), 115.16 (C-5′), 113.44 (C-2′), 103.84 (C-10), 101.85 (C-1′′), 100.06 (C-1′′′), 98.92 (C-6), 93.82 (C-8), 73.56 (C-5′′), 72.94 (C-3′′), 71.14 (C-2′′), 70.6 (C-4′′′), 70.4 (C-2′′′), 68.3 (C-5′′′), 67.98 (C-4′′), 65.18 (C-6′′), 55.94 (C-3′-O-CH_3_), 17.90 (C-6′′′). 

The compounds **GAF 3** is a diglycoside that has already been identified in: *Gomphrena martiana* Moquin (Amaranthaceae) [39]; *Blackstonia perfoliata* (L.) (Gentianaceae) aerial parts [42]; *NitrariaRetusa* (Nitrarioideae) leaves [40]; and *Calotropis procera* R. Br. (Asclepiadaceae) leaves [44]. No reports were found for the Rubiaceae family.

*Kaempferol-3-O-robinoside-7-O-rhamnoside* (robinin) [45] (**GAF 4**, Figure 2): λ_max_ 266, 346 nm. It was assigned with a molecular formula C_33_H_39_O_20_ by ESI-MS, *m*/*z* 739.2062. [M − H]^−^. ^1^H-NMR (400 MHz, DMSO-*d*_6_) *δ*: 8.10 (2H, d, *J* = 9.0 Hz, H-2′, 6′), 6.88 (2H, d, *J* = 9.0 Hz, H-3′, 5′), 6.81 (1H, d, *J* = 2.1 Hz, H-8), 6.46 (1H, d, *J* = 2.1 Hz, H-6), 5.56 (1H, d, *J* = 1.4 Hz, H-1′), 5.36 (1H, d, *J* = 7.6 Hz, H-1′′), 4.40 (1H, d, *J* = 1.7 Hz, H-1′′′), 3.09 (1H, dd, *J* = 12 Hz, 6.5, H-4′′′), 1.12 (2H, d, *J* = 6.1 Hz, H-6′′′′), 1.05 (2H, d, *J* = 6.2 Hz, H-6′′′). ^13^C-NMR (100 MHz, DMSO-*d*_6_) *δ*: 177.66 (C-4), 161.64 (C-5), 160.19 (C-4′), 157.08 (C-2), 156.04 (C-9), 133.57 (C-3), 131.11 (C-6′), 120.71 (C-1′), 115.14 (C-5′), 105.60 (C-10), 103.29 (C-1′′), 100.03 (C-1′′′), 99.73 (C-6), 98.39 (C-1′′′′), 94.88 (C-8), 73.61 (C-5′′), 72.96 (C-3′′), 71.92 (C-4′′′), 71.62 (C-4′′′), 71.10 (C-2′′, 2′′′′), 70.60 (C-3′′′), 70.43 (C-2′′′), 70.27 (C-3′′′′), 69.85 (C-5′′′′), 68.28 (C-5′′′), 68.00 (C-4′′), 17.93 (C-6′′′).

The compounds **GAF 4** is a flavone triglycoside of the well-studied has already been identified in: *Alternanthera brasiliana* (Amaranthaceae) leaves [37]; *Strychnos variabilis* (Loganiaceae) leaves; *Atropa beladona* (Solanaceae) leaves [38]; *Astragalus falcatus* Lam. (Leguminosae) flowers [45]; *Robinia pseudoacacia* (Leguminosae) leaves [46]; and *Melilotus elegans* Salzm. ex Ser. (Leguminosae) leaves [47]. No reports were found for the Rubiaceae family.

*Isorhamnetin-3-O-robinoside-7-O-rhamnoside* [35] (**GAF 5**, Figure 2): λ_max_ 255, 354 nm. It was assigned with a molecular formula C_34_H_42_O_21_ by ESI-MS, *m*/*z* 769.2220 [M − H]^−^. ^1^H-NMR (400 MHz, DMSO-*d*_6_) *δ*: 8.02 (1 H, d, *J* = 2.1 Hz, H-2′), 7.60–7.57 (1H, m, H-6′), 6.93–6.90 (1H, m, H-5′), 6.46 (1H, d, *J* = 2.2 Hz, H-8), 5.57 (1H, d, *J* = 2.1 Hz, H-1′′′′), 5.48 (1H, d, *J* = 7.7 Hz, H-1′′), 3.87 (3H, s, H-3′-O-CH_3_), 3.12–3.05 (1H, m, H-4′′′), 1.13 (3H, d, *J* = 6.1 Hz, H-6′′′′), 1.06 (3H, d, *J* = 6.1 Hz, H-6′′′). ^13^C-NMR (100 MHz, DMSO-*d*_6_) *δ*: 178.01 (C-4), 162.08 (C-7), 161.34 (C-5), 157.35 (C-9), 156.46 (C-2), 150.12 (C-4′), 147.50 (C-3′), 133.84 (C-3), 122.69 (C-6′), 121.33 (C-1′), 115.64 (C-5′), 113.87 (C-2′), 106.08 (C-10), 102.16 (C-1′′), 98.81 (C-6), 95.18 (C-8), 73.38 (C-5′′), 72.34 (C-4′′′), 71.58 (C-4′′′′), 71.05 (C-2′′), 70.73 (C-3′′′′), 70.30 (C-3′′′), 56.40 (C-3′-O-CH_3_), 18.38 (C-6′′′′), 18.34 (C-6′′′).

The compounds **GAF 5** is a triglycoside that has already been identified in *Rhazya stricta* Decaisne (Apocynaceae) leaves [35]; and *Blackstonia perfoliata* (L.) (Gentianaceae) aerial parts [42].

Only after the isolation was it possible to analyze the compounds by ^1^H- and ^13^C-NMR and to differentiate the types of sugar present, being possible to identify the final portion as galactose. In addition, it can be confirmed that the sugars are present in positions-*3* (**GAF 1**–**5**) and-*7* (**GAF 4**–**5**).

Compound **GAF 1** exhibited in vitro inhibitory activities against leukemia K562 cells in different extents [47], and cytotoxic activity against HepG-2cells.The hydroxylation pattern of the *C*-rings of the flavonoid compounds like quercetin aglycone, play an essential role in their cytotoxic activities, especially the inhibition of protein kinase antiproliferation activity [48].

*Kaempferol-3-O-robinoside* (**GAF 2**) significantly inhibited the human lymphocyte proliferation *in vitro*, to a greater extent (IC50 ≅ 25 μg mL^−1^) and were twice more active than crude extract of *Alternanthera brasiliana* [37]. The compound **GAF 2** showed radical scavenging activities when evaluated using the DPPH method. The IC50 values of the DPPH radical were 286.7 mΜ [43].

The isorhamnetin 3-*O*-robinoside (**GAF 3**) showed a protective effect against lipid peroxidation induced by H_2_O_2_ and antigenotoxic potential on human chronic myelogenous leukemia cell line K562 [49].

Flavonoids are considered as a class of natural products of high pharmacological potency but, unfortunately, many of them have a low solubility in water. We have also isolated one flavonol glycosides very soluble in water: robinin (**GAF 4**). Similar compound have been previously isolated from leaves of *Atropa belladonna* [38] but your structure have not been fully elucidated. Robinin (**GAF 4**) displayed a marked activity, inhibiting edema (38.8%) at a concentration of 0.0027 mmol/kg of body weight, four hours after injection of carrageenin [47]. **GAF 4** was also able to inhibit lymphocyte proliferation to a greater extent (IC50 ≅ 25 μg mL^−1^) and were twice more active than crude extract of *Alternanthera brasiliana* [37].

No reports about pharmacological activities were found for **GAF 5**.

This work described the first time the isolation and identification of flavonoids in leaves at *G. americana*. This is an important finding for subsequent studies aimed at the standardization of leaf extracts.

## 3. Materials and Methods 

### 3.1. Plant Material

The leaves of *Genipa americana* L., Rubiaceae, were collected in Natal City, Rio Grande do Norte State, Brazil, at coordinates lat: −6.1278 long: −35.1115 WGS22, in May 2012. The plant was identified by botanist Alan de Araújo Roque (UFRN) and a voucher specimen was deposited at the Herbarium of the Federal University of Rio Grande do Norte (UFRN), Brazil, with an identification number 12251.

The collection of the plant material was conducted under authorization of Brazilian Authorization and Biodiversity Information System (SISBIO) (process number 35017) and National System for the Management of Genetic Heritage and Associated Traditional Knowledge (SISGEN) (process number A618873).

### 3.2. Extraction and TLC Characterization

The leaves of *G. americana* was evaporated at 40 °C in a circulating air oven and powdered leaves (600 g) were extracted by maceration with ethanol:water (70:30, *v*/*v*) for five days (plant:solvent, 1.5:10, *w*/*v*; 4 L; at room temperature). Then, the organic solvent was evaporated under reduced pressure in rotary evaporator (temperature below 45 °C) and water residue was freeze-dried, obtaining the hydroethanolic extract (HE).

The HE was submitted to a liquid-liquid extraction with organic solvents in order of increasing polarity: petroleum ether (PE) (3 × 200 mL), was sequentially partitioned with chloroform (3 × 200 mL), ethyl acetate (3 × 200 mL) and *n*-butanol (3 × 200 mL). The fractions were evaporated under reduced pressure (temperature below 45 °C) and respectively afforded the PE (0.70 g), CHCl_3_ (2.9589 g), EtOAc (3.27 g), and *n*-BuOH (14.51 g) fractions of the leaves of *G. americana*.

In the phytochemical screening the HE and fractions were analyzed by TLC using aluminum sheets, coated with silica gel F254 as absorbent and chromatographed with ethyl acetate:formic acid:water: methanol (10:1.6:1.5:0.6, *v*/*v*/*v*/*v*) as mobile phase. The TLC was analyzed under 254 and 365 nm ultraviolet (UV) light and then sprayed with vanillin sulfuric acid (4%) or natural product reagent (0.5%)—NP reagent.

### 3.3. UHPLC Characterization

The samples were analyzed by ultra-high performance liquid chromatography coupled with a diode array detector (UHPLC/HPLC-DAD), model UFLC (Shimadzu, Kyoto, Japan), containing a quaternary pump system (LC-20A_3_ XR), equipped with a degasser (DGU-20A_3_), auto-sampler (SIl-20AC XR), column oven (CTO-20AC), and diode-array detectors (SPD-M20A), with *Software* LC *Solution* (Shimadzu, Kyoto, Japan) controlled system. A C_18_ column Shim-pack XR-ODS 30 × 2 mm, 2.2 µm (Shimadzu, Kyoto, Japan); a temperature of 25 ± 2 °C was used for the analysis and separation of the compounds and was achieved using a solvent system mixture of acetonitrile: acidified water (with 0.3% formic acid) as the mobile phase with a flow rate of 0.3 mL/min, and a detector was set at 254 nm.

### 3.4. HPLC-ESI-IT-MS/MS Characterization 

The hydroethanolic extracts and EtOAc and BuOH fractions were analyzed by HPLC-IT-MS/MS. UFLC (Shimadzu, Kyoto, Japan) containing two LC20AD solvent pumps, a SIL20AHT auto sampler, a SPD-M20A detector and a CBM20A system controller, coupled with an ion-trap mass spectrometer (AmaZon X, Bruker, Billerica, MA, USA). LC experiments were performed using a C_18_ column (Kromasil—250 mm × 4.6 mm × 5 μm) and the following gradient elution: solvent A: water and formic acid (0.1%, *v*/*v*); solvent B: acetonitrile; injection volume of 20 μL, and flow rate of 0.6 mL/min.

The ion-trap analysis parameters are as follows: capillary 4.5 kV, ESI in positive mode, final plate offset 500 V, 40 psi nebulizer, dry gas (N_2_) with flow rate of 8 mL/min and a temperature of 300 °C. CID fragmentation was achieved in auto MS/MS mode using advanced resolution mode for MS and MS/MS mode. The spectra (*m*/*z* 50–1000) were recorded every two seconds.

The data obtained were interpreted with the help of the following: *Metlin*, *MassBank* and *Scienfinder*.

### 3.5. Isolation of Major Compounds

According to their profiles by TLC, the EtOAc and *n*-BuOH fractions were gathered for subsequent isolation. Fractionation of the compounds of *G. americana* started with the EtOAc fraction (3.27 g), which was submitted to vacuum liquid chromatography (VLC) (10 × 15 cm) on a sintered funnel filled with silica gel 60 (0.063–0.200 mm) and eluted with *n*-hexane (50:10):methanol (50:50):water (10:100) *v*/*v*, 150 mL. This procedure yielded in 10 fractions. In this step, the fractions that showed the presence of flavonoids in TLC analysis were chosen for isolation. Fractions 8 and 9 of VLC were gathered (1.5 g) and further subjected to classical column chromatography (25 × 3 cm) in a Sephadex LH-20(GE Healthcare Bio-Science AB, Uppsala, Sweden), eluted with chloroform:water:methanol (9:0.1:0.9; *v*/*v*; 2.0 mL/min) affording 134 fractions. Fractions 61–111 were gathered (Fr. 61–111, 153 mg) and submitted to purification by HPLC (mobile phase: acetonitrile (21–23%) using a Shimadzu Shimpack ODS (H) kit, C_18_ column (200 mm × 20 mm, 5 µm), flow rate 10 mL/min and UV 254 nm, 270 nm and 340 nm) (for details, see Section 3.6). This procedure yielded compounds **GAF 1** (4.2 mg), **GAF 2** (4.0 mg) and **GAF 3** (6.0 mg).

The *n*-BuOH fraction (5.0 g) was submitted to VLC (10 × 15 cm) on silica gel 60 (0.063–0.200 mm) and eluted with gradient-mode methanol:EtOAc (0–100%; *v*/*v*). This procedure resulted in 11 fractions. Fraction 4 (1.07 g) was subjected to classical column chromatography (25 × 3 cm) on silica gel 60 (0.063–0.200 mm) and eluted with EtOAc:formic acid:water:methanol (6.5:1.4:1:1; *v*/*v*), sub-fraction 4-E showed greater intensity of phenolic compounds in analysis by TLC, being selected for isolation steps. The fraction 4-E of chromatography (1.0 g) was dissolved in methanol and chilled to 2 °C, for five days, which promoted the crystallization of a part of the sample, which was separated and named fraction 4-E.1 (crystallized, 60 mg) and fraction 4-E.2 (not crystallized, 440 mg). The sub-fraction 4-E.2 was submitted to preparative HPLC developed with water:acetonitrile (0–30 min, 18%; 10 mL/min) (for details, see Section 3.6) using C_18_ column (200 mm × 20 mm, 5 µm), and UV 254 nm, 270 nm, and 340 nm. This procedure yielded compounds **GAF 4** (10.0 mg) and **GAF 5** (9.0 mg).

### 3.6. Preparative HPLC Optimization and Analyses

In order to obtain the isolate compounds, these fractions were subjected to preparative HPLC for further purification. The preparative HPLC separations were used with a C_18_ column (200 mm × 20 mm, 5 µm); and mobile phase was selected based on the polarity of the likely compounds and the analytical HPLC conditions. Several mobile phases composed of acetonitrile (B)–water(A) in various concentrations of acetonitrile (15%, 18%, 20%, 21%, 23%, 24%, 25%, 30%) were tested. The results indicated that the best separation conditions were achieved using acetonitrile in a gradient mode (0–28 min, 21–23%) for the ethyl acetate fraction and isocratic mode (0–30 min, 18%) for *n*-BuOH fraction and a flow rate of 10 mL/min and a monitoring wavelength of 254 nm, 270 nm and 340 nm, with LabSolutions software (Shimadzu, Kyoto, Japan). Under the above conditions, a satisfactory separation of each the targeted compounds was achieved.

### 3.7. MS/MS of Isolated Compounds

The isolated compounds were analysis by MS-MS in positive and negative modes were by mass of direct infusion the microTOF II-ESI-TOF—Bruker Daltonics (Bruker, Billerica, MA, USA), with a drying gas flow rate o f4 L/min at 180 °C, nebulizer gas 0.4 bar (pressure), internal calibration standard: TFA, and syringe flow: 10 µL/min.

### 3.8. Nuclear Magnetic Resonance Spectroscopy (NMR) of Isolated Compounds

The nuclear magnetic resonance (NMR) spectra were performed on a (Bruker, Billerica, MA, USA) (400 MHz for ^1^H and 100 MHz for ^13^C) and chemical shifts are given in ppm relative to residual DMSO-*d*_6_ (2.5), and to the central peak of the triplet related to DMSO-*d*_6_ carbon (39.5 ppm).

## 4. Conclusions

Through characteristic fragmentation patterns of substances obtained by MS/MS data, 13 compounds were identified. The flavonoids were isolated and identified.

In contrast to literature, which describes mainly the presence of iridoids for the fruit and leaf extracts of *G. americana*, in this paper the leaves showed to be rich in *O*-*glycosidic* flavonoids. The isolation was carried out in few steps and allowed the identification of five flavonoids glycosides not described until this moment to the *G. americana* leaf extract. These flavonoids **GAF 1**, **3**, **4**, and **5** were identified for the first time in the Rubiaceae family and flavonoid **GAF 2** is unknown for genus *Genipa*. All flavonol aglycones have sugars units, attached only at the 3-position. The similarities of the compounds of five chemical structures suggest a common biosynthetic pathway in this species. The present article was conducted to evaluate the chromatograph profiles of the leaf extract from *Genipa americana*, to be used in future to the quality control for this species. It can also be suggesting that flavonoids identified for this species may be associated, at least in part, to pharmacological properties of the plant. 

## Figures and Tables

	**R1 (position 3′)**	**R2 (position 2′′)**	**R3 (position 7′)**
**GAF 1**	Robinoside	OH	H
**GAF 2**	Robinoside	H	H
**GAF 3**	Robinoside	OCH_3_	H
**GAF 4**	Robinoside	H	Rhamnose
**GAF 5**	Robinoside	OCH_3_	Rhamnose

**Table 1:** Phytochemical profile of leaf extract from *G. americana* obtained by HPLC-IT-ESI-MS/MS. For conditions, please see Section 3.4.

Peak nº	*Rt* (min)	*m*/*z*	*m*/*z* MS^2^	*m*/*z* MS^3^	LC-DAD	Molecular Formula	Compound (MM)
		Pos/Neg	Pos/Neg	Pos/Neg	λ_max_ [nm]		
1	5.2 ^a^	-/342	-/179	-/-	-	C_16_H_22_O_8_	Coniferin (342)
2	24 ^b^	559/535, 571^-^	-/535	373, 210	-	C_22_H_32_O_15_	Asystasioside D (536)
3	25.1 ^a^	397/373, 409	379, 217/210	172/166, 148, 122	-	C_16_H_22_O_10_	Geniposidic acid (374)
4	26.1 ^a^	-/357, 393	-/194	-/-	-	C_16_H_25_O_9_	Tarenoside (358)
5	26.9 ^b^	-/375	-/213	-/169, 125	-	C_16_H_24_O_10_	Loganic acid (376)
6	33.4 ^a,b^	355/353	162/190	-/-		C_16_H_18_O_9_	Chlorogenic acid (354)
7	48.8 ^a,b,c^	741/739^-^	595/593	433, 287/285	266, 346	C_33_H_39_O_20_	Kaempferol-3-*O*-hexoside-deoxyhexoside-7-*O*-deoxyhexoside (740)
8	50.1 ^a,b^	771/769	625, 463/623	317/315	255, 354	C_34_H_42_O_21_	Isorhamnetin-3-*O*-hexoside-deoxyhexoside-7-*O*-deoxyhexoside (770)
9	51.4 ^a,b^	611/609^-^	465/301	303/-	255, 366	C_27_H_30_O_16_	Quercetin-3-*O*-hexoside-deoxyhexoside (610)
10	51.7 ^b^	-/515	-/353, 190, 178	-/-	-	C_25_H_24_O_12_	1,3-Di-*O*-caffeoylquinic acid (516)
11	52	519, 373, 211/517, 371^-^	-/-	-/-	-	C_22_H_30_O_14_	Teneoside A (518)
12	53.9 ^a,b^	595/593	2834.7/449, 288	-/-	265, 355	C_27_H_30_O_16_	Kaempferol-3-*O*-hexoside-deoxyhexoside (594)
13	55.2 ^a,b^	625/623	479/315	317/-	255, 370	C_28_H_33_O_16_	Isorhamnetin-3-*O*-hexoside-deoxyhexoside (624)

^a^ = HE; ^b^ = Fr. EtOAc; ^c^ = Fr. *n*-BuOH; *Rt*: retention time; MM: molecular mass.
